# Multi-model-based validation of multi-environment trial results in horse gram (*Macrotyloma uniflorum* Lam. Verdc.)

**DOI:** 10.1038/s41598-025-25093-2

**Published:** 2025-11-21

**Authors:** Sudhagar Rajaprakasam, Sumaiya Sulthana Jafarullakhan, Vaishnavi Vijayakumar, Naaganoor Ananthan Saravanan, Sivakumar Rathinavelu, Balaji Kannan, Vanniarajan Chockalingam, Raveendran Muthurajan, Selvaraju Kanagarajan

**Affiliations:** 1 Office of the Dean (Agriculture), TNAU, Coimbatore, India; 2 Centre for Plant Breeding and Genetics (CPBG), Tamil Nadu Agricultural University (TNAU), Coimbatore, India; 3 Centre for Plant Breeding and Genetics (CPBG), TNAU, Coimbatore, India; 4 Sugarcane Research Station, TNAU, Melalathur, Vellore, Tamilnadu India; 5 Department of Crop Physiology, Crop Management Studies, TNAU, Coimbatore, India; 6 Department of Physical Sciences and Information Technology, AEC and RI, TNAU, Coimbatore, India; 7 Anbil Dharmalingam Agricultural College and Research Institute, TNAU, Trichy, India; 8 Directorate of Research, TNAU, Coimbatore, India; 9 Department of Plant Breeding, Swedish University of Agricultural Sciences, P.O. Box 190, Lomma, 234 22 Sweden

**Keywords:** BLUP, Horse gram, MTSI, WAASB, YREM, Plant breeding, Plant genetics

## Abstract

Analysis of genotype-by-environment interactions (GEI) is critical for evaluating the yield and stability of genotypes in multi-environment experiments (METs). Either fixed models (AMMI and GGE biplots) or random effect models (Linear mixed models: LMM) are utilised to estimate GEI. From our preliminary METs using fixed models, a few potential horse gram mutant genotypes (G1, G3, G25, & G27) were identified. To validate this finding, confirmatory METs were performed during the 2023 (E1, E2, & E3) and 2024 (E4, E5, & E6) cropping seasons using LMM and the yield relative to the environmental maximum (YREM) index at pulses experimental farm, Coimbatore (E1 & E4), sugarcane research station, Melalathur, Vellore (E2 & E5), and farmer’s field, Krishnagiri (E3 & E6). The traits positively and significantly correlated with yield were utilized for LMM and YREM analyses. The YREM value ranged from 0.55 to 1.00. The genotype G1 exhibited the highest YREM of 1.00, indicating that the crossover GEI effect did not affect its yield potential. After the likelihood ratio test (LRT), BLUP analysis was performed and identified the promising genotypes G1, G3, G22, G25, G27, and G8 based on higher predicted mean values. The genetic variance components (GVC) estimation showed low R² GEI (0.056 to 0.539), high ‘As’ (0.993 to 0.785), low to high r_ge_ (0.127 to 0.758), and medium to high H^2^ (0.068 to 0.863). The WAASB biplot identified potential genotypes (G1, G8, G27, G22, and G3) based on yield potential and stability. The multi-trait selection index (MTSI) recognized the genotypes G1 (1.192), G22 (1.250), G3 (1.319), and G8 (1.841) as superior performers. The combined YREM and LMM results revealed the significant breeding potential of G1, G3, G22, G27 and G8 for exploitation, while G22, G25, and G8 exhibited method-specific superiority. The selection differential analysis affirms the superiority of these genotypes for the tested traits (NC-25.70%, NPC-14.20%, NP-28.80%, NS-9.94%, YD-30.40%, & DM-0.05%). In conclusion, the validation METs reveal a slight variation among the findings between fixed and random effect models, emphasising the importance of integrating multiple analytical approaches for an effective GEI analysis in horse gram.

**Supplementary Information:**

The online version contains supplementary material available at 10.1038/s41598-025-25093-2.

## Introduction

Grain legumes provide essential dietary protein to millions worldwide and play a crucial role in global food security. Globally, legumes cover approximately 959.68 lakh hectares with a production of 973.92 lakh tonnes^1^. Low input requirements, the ability to enrich the soil with nitrogen through root nodules, the capacity to reduce CO_2_ emissions^2^ and carbon-enriched root exudates make legume cultivation a sustainable and environmentally beneficial choice for agriculture. India ranks first globally in both legume area and production, accounting for 38% of the world’s area and 28% of global production.

In India, legumes are cultivated on an area of 361.11 lakh hectares, producing 276.69 lakh tonnes with a productivity of 766 kg per hectare^1^. Among the legumes, Horse gram (*Macrotyloma uniflorum* (Lam.) Verdc.) is a short-day, photosensitive, and *Rabi* season legume. It is renowned for its exceptional nutritional qualities^3^, stress tolerance^4^, soil fertility enhancement, and capacity to produce nutrient-rich fodder^5^. In India, it covers about 348.16 thousand hectares, resulting in a production of 226.21 thousand tonnes and a productivity of 650 kg per hectare (www.indiastat.com). It contains good levels of protein (18–29%), essential amino acids, and some important vitamins. It’s also low in fat and contains high levels of tannins and polyphenols, offering significant medicinal and therapeutic benefits^6^. However, the development of promising cultivars through conventional hybridization methods is limited by constraints like a lack of variability at morphological and molecular levels, long maturity duration, asynchronized maturity, indeterminate growth habit, and photosensitivity^5^. Induced mutagenesis, on the other hand, has been recognized as a promising approach for generating variability in economically significant traits^7^.

The selection of superior genotypes within this variation depends on effectively exploiting the introduced genetic diversity. Alongside identifying good-yielding genotypes, stable performance of the identified genotype(s) is also an essential criterion. In any plant breeding program, substantial effort and resources are devoted to evaluating numerous genotypes across various environments through multi-environment trials (METs)^8^. These METs help in selecting genotypes with stable performance over time and across locations.

The primary challenge in analysing METs lies in interpreting the genotype-environment interaction (GEI), since genotypes do not exhibit uniform performance across environments. Exploring GEI often involves employing the Additive Main-effects and Multiplicative Interactions (AMMI) and Genotype and Genotype-Environment Interaction (GGE) models^9^.

To identify stable horse gram genotypes from the pool of promising genotypes evolved through an induced mutagenesis project, we performed a stability analysis by using AMMI and GGE models^10^ in the 2021 and 2022 cropping seasons. The effectiveness of these models has been thoroughly demonstrated by various researchers, including Vaishnavi et al.^11^ in horse gram, Azam et al.^12^ in chickpeas, Kebede and Getahun^13^ in groundnut, and Mahalingam et al.^14^ in mung bean. However, these methods are fixed-effect models, where genotypes and environments are considered as fixed effects, and the random variation in GEI may not be captured fully. This leads to a biased estimation of genotype stability. Further, in METs, weather vagaries influence crop yield significantly, and advancements in analytical methodologies necessitate utilizing multiple methods to ascertain GEI effectively. Recent research on METs has increasingly adopted linear mixed model (LMM) approaches for stability analysis, as these models can more effectively accommodate random effects in both genotypes and environments, improving predictive accuracy and handling complex GEI^8^. Studies by Daba et al.^15^, Olivoto et al.^8^, Hassani et al.^16^, and Koundinya et al.^17^ provide evidence that LMM approaches offer superior, accurate predictive results. Despite the growing importance of stability assessment in crop breeding, such reports on horse gram genotypes using LMM approaches are very few.

Identification of potential genotypes in METs based on their mean performances is traditionally followed. However, Yan^18^ used the yield relative to the environmental maximum (YREM) index in wheat for a comparative analysis of the yielding potential of test genotypes, and Ashwini et al.^19^ utilized it in horse gram MET. YREM estimates genotypic performance after nullifying environmental effects.

In light of this background, the present study was undertaken to validate our previous findings from METs conducted in the 2021 and 2022 cropping years involving AMMI and GGE^10^. The cross-validation METs were conducted in the 2023 and 2024 cropping years, utilizing LMM and YREM approaches. The LMM approach included (i) Best Linear Unbiased Prediction (BLUP) estimation, (ii) the Weighted Average of Absolute Scores (WAAS)^8^, (iii) WAASBY index, and (iv) Multi-Trait Stability Index (MTSI)^20^. These multi-year and multi-method strategies aimed to provide a comprehensive assessment of yielding potential and stability of horse gram genotypes across diverse environments.

## Results and discussion

The poor man’s legume, horse gram, is predominantly cultivated in rainfed areas; alas, its genetic variability is narrow^6^. Mutation techniques have introduced considerable genetic diversity for yield and growth traits^21^. To ascertain the breeding potential of these mutants METs involving AMMI and GGE biplot techniques were utilized^10^. To cross-validate these findings, the current METs involving YREM and LMMs were performed. The purpose of integrating various methodologies is to capture different dimensions of GEI. LMM-based BLUPs provide precise estimates of genotypic effects, WAASB and MTSI integrate stability with yield and multiple traits, and YREM quantifies the crossover interaction. Hence, this multi-model method strategy is used in these experiments to capture a comprehensive and cross-validated assessment of the yielding potential and stability of horse gram mutant genotypes. Reports combining all these models to assess the breeding potential of horse gram are yet to be published.

### Weather parameters

Horse gram is grown purely as a rainfed crop; a slight variation in the rainfall or day length significantly influences the vegetative, phenology and reproductive phases^22^. Horse gram is a typical short-day plant that requires relatively low temperature, dew, and intermittent rainfall for better performance. Changes in any one of these factors influence the yield potential. Therefore, the important weather parameters like temperature (maximum and minimum-°C), relative humidity (%), and rainfall (mm) for the cropping duration (*Rabi* season: September to February) for the two METs (MET 1: 2021 and 2022; MET 2: 2023 and 2024) are analysed. Considerable variations for these environmental factors are observed (Supplementary Fig. S1), thereby providing chances to assess the genome plasticity of the genotypes in these varied conditions. The genotypic performances in each environment for ten traits were recorded and utilized for performing correlation studies to delineate the most relevant traits associated with yield.

### Pearson’s correlation analysis

Pearson’s correlation analysis reveals the relationships between traits and aids in formulating selection criteria for improving multiple traits simultaneously^23^. Understanding these interrelationships is crucial for applying LMM-based techniques like MTSI. Therefore, the correlation analysis was performed between ten traits whose mean values were arrived at by considering the six distinct study environments (Supplementary Fig. S2). Traits NC (0.83), NS (0.79), NPC (0.76), and NP (0.59), and expressed significant and positive associations with YD signifying their role in horse gram yield improvement. These results are aligned with studies of Vaishnavi et al.^24^ in horse gram, Takele et al.^25^ in lentils, Nabati et al.^26^ in chickpeas, and Mofokeng et al.^27^ in cowpeas. The trait DM (0.08) expressed a non-significant but positive correlation with YD. Given their correlation with yield, the traits NC, NS, NPC, NP, DM, and YD were further selected for stability analyses. Although DM showed a non-significant but positive correlation with YD, it was included in the stability analyses due to its potential agronomic relevance and role in influencing yield under specific environmental conditions. The traditional horse gram genotypes are photosensitive and possess an indeterminate growth habit, and therefore DM increases the possibility of the number of pod-bearing clusters as the age of the crop increases and thus influences the YD.

### Mean performance studies

The mean values for the six traits earmarked above were compared with the parent PAIYUR 2 across study environments (Table 1). The boxplot illustrating the distribution of mean values across environments is provided in Supplementary Fig. S3. Of all the genotypes analysed, G1 possessed the highest average seed yield of 1335.28 kg/ha, followed by G3 with 1245.03 kg/ha while the parent PAIYUR 2 (G30), recorded a yield of 925.57 kg/ha. The plant breeding programs employed in any crop are primarily targeted to enhance the yield of the offspring considerably. The induced mutagenesis techniques applied in the horse gram from the cropping season 2017 to 2024 evolved a few promising horse gram genotypes with enhanced yielding potential than the parent PAIYUR 2, whose further experimentations can help in sustaining food/nutritional security in the rainfed agricultural ecosystems of India.

**Table 1:** Mean performance and YREM values of horse gram mutant genotypes tested across six environments (Average values of six environments ± SE).

Genotypes	Genotype’s names	Biometrical traits						Average YREM	YD&YREM-based genotype rank
		DM	NC	NP	NPC	NS	YD		
G1	TNAU-HG-007	123.69 ± 0.02	204.61 ± 3.30	535.07 ± 3.27	4.16 ± 0.16	6.28 ± 0.09	1335.28 ± 3.51	1.00	1
G2	TNAU-HG-031	121.63 ± 0.18	122.91 ± 0.04	397.31 ± 15.78	3.14 ± 0.07	5.44 ± 0.10	852.79 ± 24.18	0.64	20
G3	TNAU-HG-070	122.41 ± 0.40	205.19 ± 0.50	518.81 ± 6.99	3.90 ± 0.03	5.89 ± 0.15	1245.03 ± 1.97	0.93	2
G4	TNAU-HG-034	123.66 ± 0.33	186.02 ± 9.96	397.68 ± 3.37	3.12 ± 0.38	5.51 ± 0.32	1051.19 ± 30.34	0.79	7
G5	TNAU-HG-019	120.37 ± 1.57	169.59 ± 2.53	444.75 ± 7.45	3.61 ± 0.00	5.32 ± 0.21	864.61 ± 2.74	0.65	17
G6	TNAU-HG-027	119.89 ± 0.18	131.54 ± 2.60	423.53 ± 7.01	3.56 ± 0.04	5.33 ± 0.19	916.89 ± 22.29	0.69	12
G7	TNAU-HG-049	124.45 ± 0.15	169.68 ± 1.79	354.73 ± 1.51	3.34 ± 0.01	5.96 ± 0.04	908.03 ± 14.48	0.68	14
G8	TNAU-HG-018	121.53 ± 0.31	193.73 ± 4.04	521.47 ± 6.05	3.75 ± 0.00	5.84 ± 0.17	1122.83 ± 3.80	0.84	6
G9	TNAU-HG-062	120.92 ± 0.07	165.18 ± 2.00	389.51 ± 3.82	3.63 ± 0.09	5.02 ± 0.05	818.04 ± 11.09	0.61	25
G10	TNAU-HG-057	121.96 ± 1.51	170.32 ± 3.13	448.20 ± 0.43	3.29 ± 0.13	5.48 ± 0.18	886.31 ± 0.48	0.66	15
G11	TNAU-HG-073	120.90 ± 0.65	124.84 ± 1.27	409.58 ± 2.80	3.55 ± 0.21	5.35 ± 0.31	854.22 ± 2.93	0.64	19
G12	TNAU-HG-083	120.80 ± 0.29	145.97 ± 2.44	351.02 ± 0.17	3.59 ± 0.14	5.44 ± 0.23	909.15 ± 10.21	0.69	13
G13	TNAU-HG-036	120.48 ± 0.31	170.57 ± 1.77	325.07 ± 1.43	3.26 ± 0.04	5.24 ± 0.32	863.82 ± 12.83	0.65	16
G14	TNAU-HG-030	118.66 ± 1.05	126.23 ± 0.74	417.03 ± 5.17	3.28 ± 0.10	4.96 ± 0.01	729.49 ± 11.47	0.55	30
G15	TNAU-HG-082	121.10 ± 0.45	198.09 ± 1.95	320.01 ± 1.05	3.61 ± 0.11	4.86 ± 0.14	953.69 ± 7.41	0.71	10
G16	TNAU-HG-025	119.56 ± 0.61	142.57 ± 0.58	428.23 ± 5.63	3.16 ± 0.13	5.18 ± 0.11	804.59 ± 5.23	0.63	21
G17	TNAU-HG-053	118.50 ± 0.26	115.91 ± 1.85	380.95 ± 0.98	3.19 ± 0.15	5.09 ± 0.04	790.24 ± 1.57	0.60	27
G18	TNAU-HG-016	118.68 ± 1.09	127.36 ± 0.62	458.40 ± 0.36	3.36 ± 0.14	4.96 ± 0.32	840.80 ± 11.38	0.63	22
G19	TNAU-HG-071	123.53 ± 0.13	122.58 ± 0.88	349.50 ± 1.03	3.49 ± 0.17	5.39 ± 0.11	825.18 ± 1.94	0.62	23
G20	TNAU-HG-075	120.10 ± 0.07	118.10 ± 3.13	377.86 ± 0.65	3.05 ± 0.00	4.92 ± 0.11	784.59 ± 2.06	0.61	26
G21	TNAU-HG-065	122.96 ± 0.55	114.76 ± 4.00	340.12 ± 2.07	2.98 ± 0.02	4.84 ± 0.02	758.92 ± 9.64	0.58	28
G22	TNAU-HG-089	120.63 ± 0.03	199.20 ± 1.58	521.97 ± 5.72	3.93 ± 0.01	5.76 ± 0.19	1212.73 ± 1.79	0.91	4
G23	TNAU-HG-032	123.66 ± 0.75	156.34 ± 4.01	418.26 ± 0.07	3.47 ± 0.00	5.55 ± 0.24	863.36 ± 12.71	0.65	18
G24	TNAU-HG-003	121.51 ± 0.56	122.88 ± 3.77	357.57 ± 4.06	3.09 ± 0.00	5.11 ± 0.02	810.48 ± 4.74	0.61	24
G25	TNAU-HG-081	114.66 ± 1.96	194.86 ± 3.07	485.29 ± 12.66	3.63 ± 0.18	6.28 ± 0.10	1217.08 ± 7.21	0.91	3
G26	TNAU-HG-011	120.59 ± 1.95	137.02 ± 1.25	391.01 ± 3.29	3.23 ± 0.00	5.39 ± 0.00	768.52 ± 5.18	0.58	29
G27	TNAU-HG-039	119.98 ± 0.44	199.13 ± 2.32	517.42 ± 3.08	3.63 ± 0.12	5.50 ± 0.29	1160.29 ± 8.22	0.87	5
G28	TNAU-HG-076	122.71 ± 0.63	187.07 ± 4.26	309.85 ± 4.76	3.48 ± 0.03	5.36 ± 0.48	1020.50 ± 10.37	0.76	9
G29	TNAU-HG-044	125.96 ± 0.00	177.73 ± 5.79	418.28 ± 22.30	3.39 ± 0.19	5.67 ± 0.33	1048.48 ± 61.66	0.79	8
G30	PAIYUR2	126.58 ± 0.09	189.86 ± 4.33	204.26 ± 3.99	3.53 ± 0.09	5.20 ± 0.20	925.57 ± 3.36	0.69	11
	Mean	121.40	159.66	407.09	3.45	5.40	938.09	-	-
	Minimum	114.66	114.76	204.26	2.98	4.84	729.49	-	-
	Maximum	126.58	205.19	535.07	4.16	6.28	1335.28	-	-
	SD	2.38	31.70	75.10	0.28	0.38	163.70	-	-
	CV(%)	1.96	19.85	18.45	8.12	7.07	17.45	-	-

DM -Days to maturity, NC -Number of clusters/plant, NP -Number of pods/plant, NPC – Number of pods/cluster, NS -Number of seeds/pod, YD -Yield/hectare, SE -Standard Error, SD -Standard deviation, CV -Co-efficient of variation, YREM -Yield relative to the environmental maximum.

### Evaluation of genotypes based on the YREM index

The YREM index was calculated to ascertain the relative yield performance of 30 horse gram genotypes across environments in the GEI perspective, and the average YREM values were arrived at (Table 1). YREM is a standardized estimate of genotype performance where the environmental main effect is nullified^18^. Unlike conventional indices such as the superiority index that depend directly on absolute yield values, YREM provides a relative and intuitive measure of a genotype’s performance across environments. It is independent of the number of genotypes tested and directly captures the extent of crossover GEI. A higher YREM index indicates a lower magnitude of crossover GEI, suggesting that the genotype experiences minimal yield reduction even in the presence of GEI. This makes YREM particularly useful in identifying genotypes with broad adaptability under variable environments.

In the current study, the average YREM value for genotype G1 is 1.00, which signifies that G1 experienced no yield loss. The YREM also identified other potential genotypes G3, G25, G22, and G27, whose average YREM values are 0.93, 0.91, 0.91, and 0.87, respectively. The yield losses in these genotypes are 7, 9, 9, and 13%, respectively. Earlier, Ashwini et al.^19^, Bomma et al.^28^, and Spoorthi et al.^29^ used the YREM index for analysing the cross-over GEI effects in horse gram, pigeon peas and dolichos bean, respectively.

### Studies on AMMI-arrived analysis of variance

Evaluating the variation among genotypes and their interactions with the environment(s) is crucial for understanding the impact of environmental conditions on a test genotype’s performance in METs. The AMMI-arrived ANOVA in METs provides a clear insight into interaction effects, which is essential for understanding the dimensionality and impact of interactions. It effectively captures and illustrates the complexity of the GEI, offering a foundational understanding of methods to be applied in analysing METs like BLUP^8^. The results of the AMMI-arrived ANOVA (Table 2.) showed significant variations for G, E and GEI for most of the experimented traits. The source of variation contributed by genotypes was higher than the environment for the traits YD (91.81%), NP (86.36%), and NC (83.11%). Whereas, the interaction effects are higher for NPC (50.42%), DM (45.16%) and NS (43.76%). It emphasises that the genetic effects are the major basis of variation however, the contribution of E and GEI needs to be studied further to understand the trait expression. Also, the significances of GEI and E suggest employing BLUP in analysing the current METs. From these results, it is understandable that some genotypes outperform others across environments due to their strong genetic potential. This result aligns with the earlier research outcomes of Danakumara et al.^30^ in chickpeas, Greveniotis et al.^31^ in faba beans, and Hanume et al.^32^. in sweet potato.

**Table 2:** AMMI arrived ANOVA (Analysis of Variance) for yield and its attributing traits in horse gram mutant genotypes.

Source of variation	Df	DM		NC		NP		NPC		NS		YD	
		MSS	%SS	MSS	%SS	MSS	%SS	MSS	%SS	MSS	%SS	MSS	%SS
Environment (E)	5	128.90	2.87	742.28	0.29	12754.86**	0.93	1.77**	4.59	9.05**	12.12	48788.31**	0.83
Genotypes (G)	30	182.36**	23.61	36172.30**	83.11	203063.10**	86.36	2.82	42.38	0.41**	40.83	12081.14**	91.81
Interactions (GEI)	29	69.74**	45.16	1371.42**	15.75	5489.95**	11.67	0.67	50.42	5.26**	43.76	927671.98**	6.11
PC1	145	98.25**	-	3012.26**	-	18660.71**	-	1.25**	-	1.13**	-	12354.15**	-
PC2	33	81.96**	-	2592.43**	-	3548.30**	-	0.75**	-	2.10**	-	26415.56**	-
PC3	31	70.10**	-	358.21**	-	1803.19**	-	0.64**	-	1.65**	-	16660.16**	-
PC4	29	64.66**	-	245.02**	-	418.20**	-	0.36**	-	0.72**	-	9030.85**	-
PC5	27	22.05**	-	83.31	-	266.32**	-	0.16**	-	0.60**	-	4167.16**	-
Residuals	25	37.20	-	69.41	-	439.93	-	0.03	-	0.24	-	1150.60**	-
Total	870	52.99	-	1242.94	-	6533.94	-	0.26	-	0.07	-	2373.68**	-

DM - Days to maturity, NC -Number of clusters/plant, NP -Number of pods/plant, NPC – Number of pods/cluster, NS -Number of seeds/pod, YD -Yield/hectare.

df- degree of freedom, MSS-Mean sum of square, %SS- % of variation explained by sum of squares.

**Significance at 1% level.

### BLUP analysis

BLUP analysis delivers the most accurate and unbiased predictions of genotypic performance by integrating information from both fixed and random effects. BLUP analysis is done based on the LRT significance for GEI variances as suggested by Olivoto et al.^8^. In the current experiment, the LRT significance was observed for G and GEI (Table 3.) for all the traits, suggesting the applicability of BLUP.

**Table 3:** Estimation of GVC (genetic variance components) using mixed models for biometrical traits in horse gram.

Likelihood ratio test						
	DM	NC	NP	NPC	NS	YD
GEN	3.13**	966.70**	5488.00**	0.06**	0.114**	25425.00**
GEN: ENV	5.42**	217.00**	841.70**	0.11**	0.175**	1663.00**
Error	37.20	69.41	439.90	0.03	0.074	2374.00
Genetic variance components (GVC) estimation						
R^2^ GEI	0.118	0.173	0.124	0.539	0.481	0.056
A_S_	0.785	0.981	0.986	0.873	0.886	0.993
r_ge_	0.127	0.758	0.656	0.773	0.701	0.412
H^2^	0.068	0.771	0.810	0.302	0.314	0.863

Significance level- 1%‘**’.

DM -Days to maturity, NC -Number of clusters/plant, NP -Number of pods/plant, NPC – Number of pods/cluster, NS-Number of seeds/pod, YD -Yield/hectare, R² GEI- Co-efficient of determination of GEI, As- Selection accuracy, r_ge−_- the genotype-environment correlation, H^2^- Heritability, GEN-genotypes, GEI: ENV- genotype environment interaction.

### Estimation of predicted mean values from BLUP analysis

**Fig. 1:**
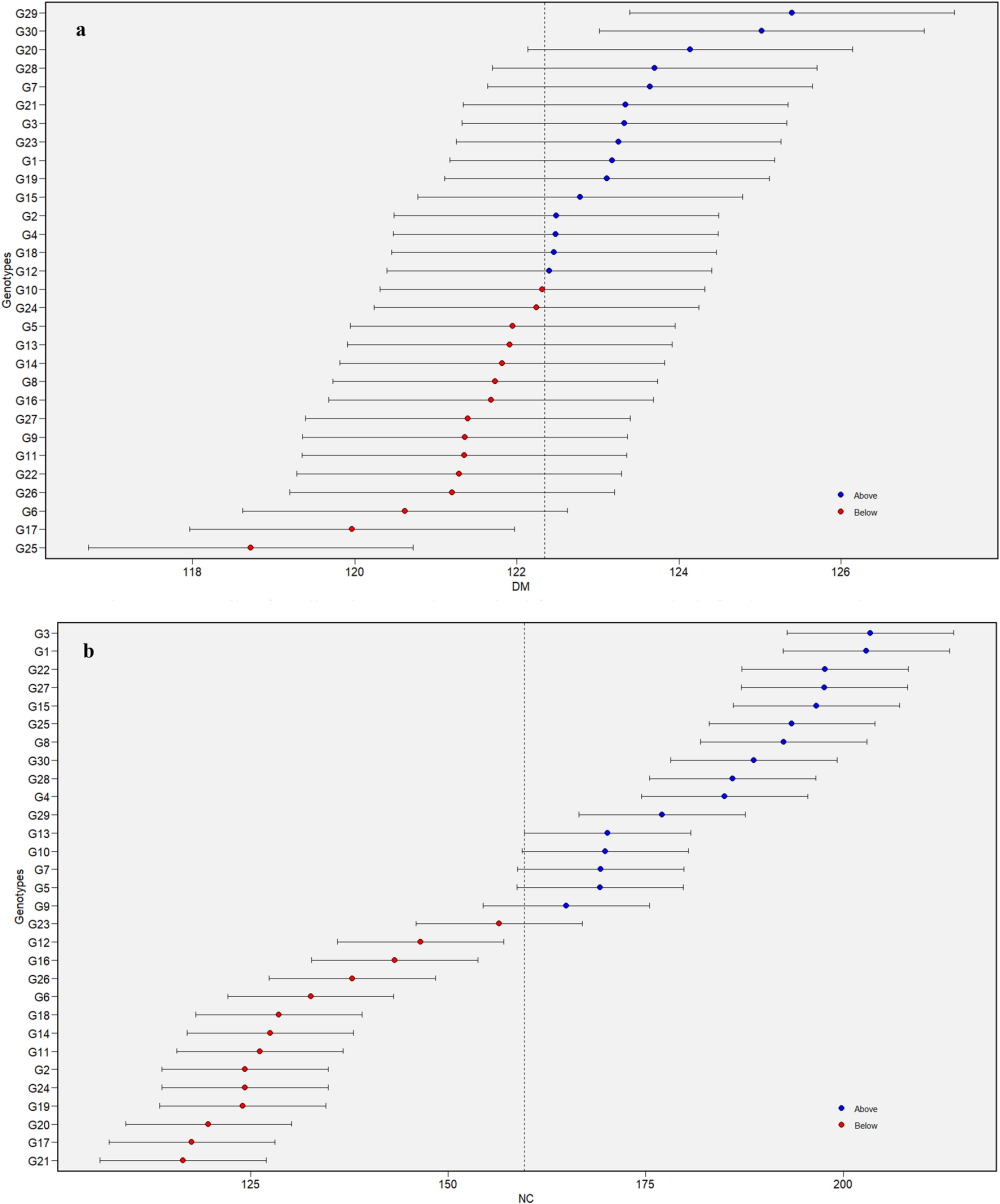

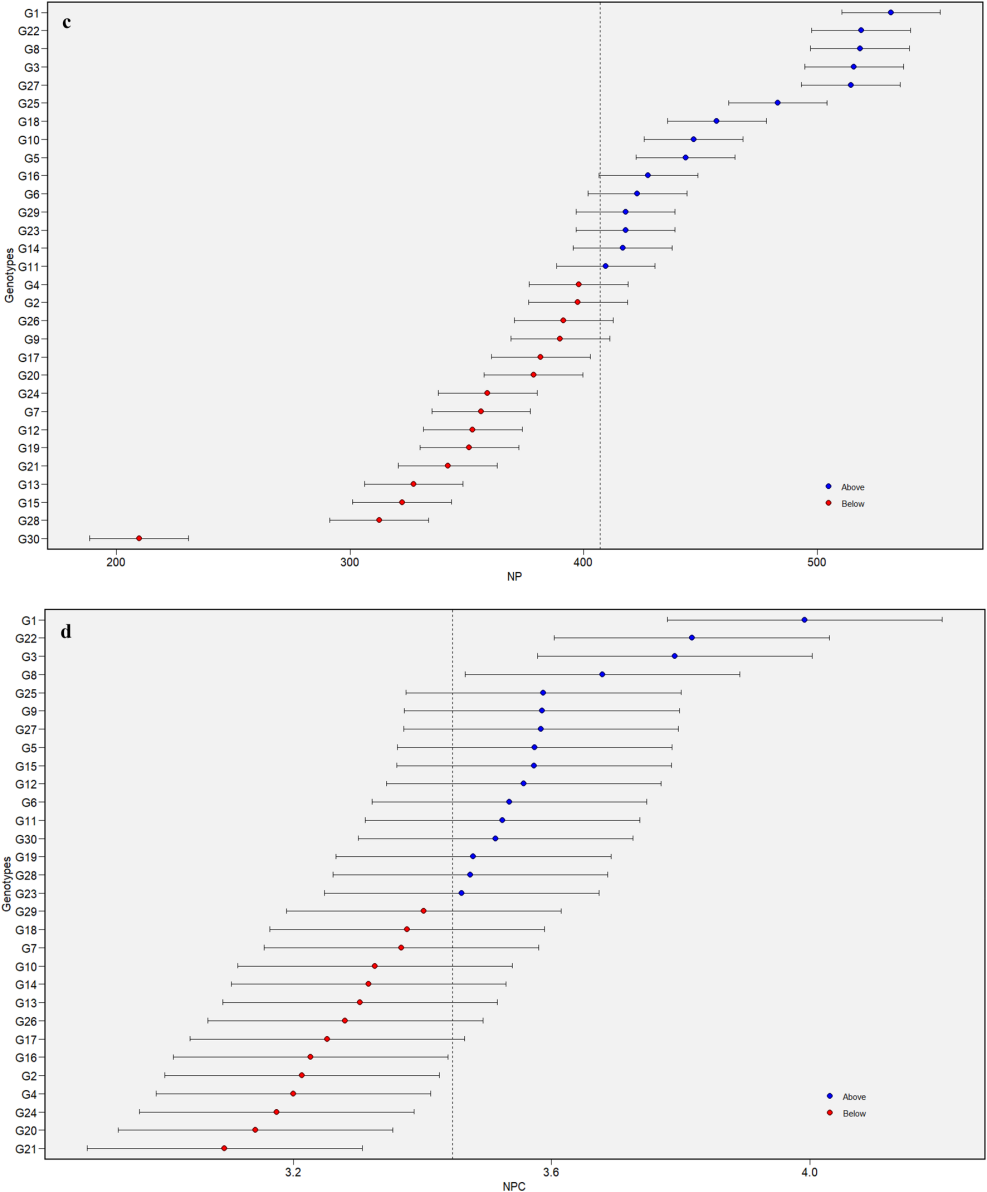

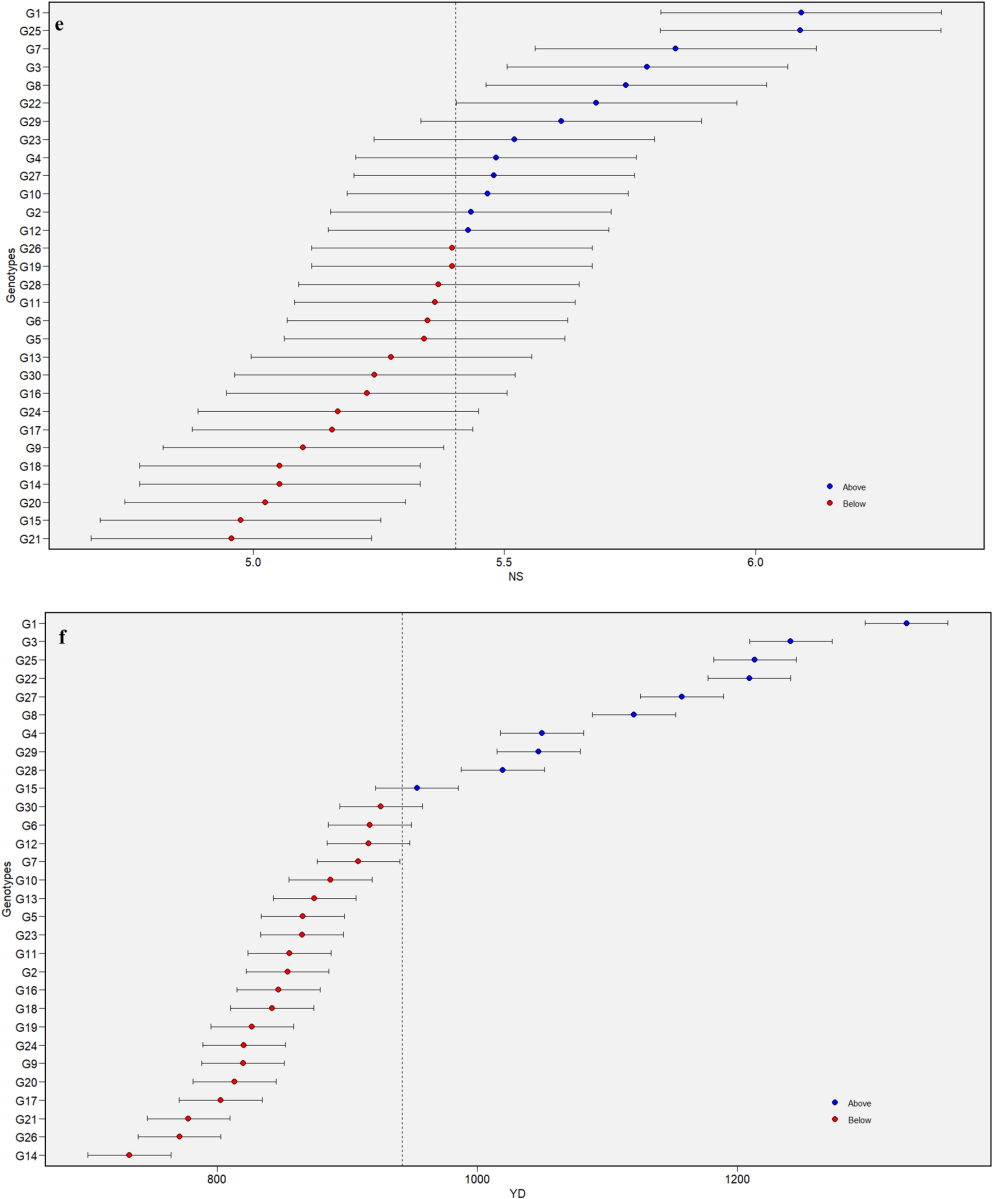
Details of predicted mean values arrived from BLUP analysis for days to maturity -DM (**a**) in horse gram mutant genotypes. Details of predicted mean values arrived from BLUP analysis for number of clusters/plant -NC (**b**) in horse gram mutant genotypes. Details of predicted mean values arrived from BLUP analysis for number of pods/plant – NP (**c**) in horse gram mutant genotypes. Details of predicted mean values arrived from BLUP analysis for number of pods/cluster – NPC (**d**) in horse gram mutant genotypes. Details of predicted mean values arrived from BLUP analysis for number of seeds/pod– NS (**e**) in horse gram mutant genotypes. Details of predicted mean values arrived from BLUP analysis for yield/hectare– YD (**f**) in horse gram mutant genotypes.

The predicted mean values for a specific trait can be derived from the BLUP estimates using a mixed model approach that incorporates both random and fixed effects. The BLUP method ensures highly accurate genotypic predictions and complements AMMI by addressing gaps in its analysis, thereby offering a more comprehensive understanding of genotypic performance across locations^33^. Further, the BLUP-based predictions are accurate and unbiased, reflecting the true genotypic values^34^. The results of the genotypic predictions by BLUP are shown in (Fig. 1a-f), where the predicted mean value of traits is plotted on the x-axis and genotypes on the y-axis. The genotypes plotted above the predicted average for a trait are considered promising. Accordingly, a total of 15, 16, 15, 16, 13 and 10 genotypes proved promising for DM, NC, NP, NPC, NS and YD, respectively. By considering the sustained superiority of all the traits, the genotypes G1, G3, G22, G25, G27, and G8 offer the scope of potential for utilization to maximize the genetic gain over time. Earlier, Alam et al.^33^ in sweet potatoes and Koundinya et al.^17^ in cassava predicted the means of genotypes using BLUP.

### Estimation of genetic variance components (GVC) parameters from mixed models

In this experiment, GVC parameters R^2^GEI, As, r_ge_, and H^2^ were estimated to assess the genotypic and interaction variances, heritability of genotypic means, and genotype-environment correlations (Table 3.). Estimating GVC parameters is important in evaluating the genotypic evaluation in MET and also crucial from a breeding perspective, as they guide plant breeders in formulating effective strategies to achieve genetic gain(s).

### Analysis of R^2^GEI

R^2^GEI estimates the magnitude of GEI variance explained by BLUP. Low R^2^GEI is observed for all the traits (0.056 to 0.539), indicating that the traits are largely influenced by either genotype or environmental effects. This also implies that the effect of GEI has a relatively minimal impact on total variation. This result confirms the AMMI-arrived ANOVA where a strong genotype-influenced source of variation was observed (Table 2.).

### Analysis of selection accuracy (As)

It estimates the correlation between the true and predicted genetic values^8^ of test genotypes. In this study, the ‘As’ values for the tested are high; DM (0.785), NC (0.981), NPC (0.873), NP (0.986), NS (0.886), and YD (0.993) referring to a strong relationship between the predicted and observed values for these traits, which is useful for selecting superior genotypes. Comparable results for ‘As’ were also reported by Taleghani et al.^35^. in sugar beet.

### The genetic correlation between environments (r_ge_) and heritability (H^2^) analysis

The r_ge_ provides insight into the stable expression of the traits across diverse environments. Selecting genotype(s) possessing traits with high r_ge_ will be more rewarding. The trait NPC had the highest r_ge_ of 0.773 (Table 3.), followed by NC (0.758), NS (0.701), NP (0.656), YD (0.412), and DM (0.127), indicating that selection of stable and good-yielding genotypes based on these traits is feasible. On the contrary, traits with the lower r_ge_ suggest that still more precise genetic information is required to employ them in selecting stable and superior genotypes. Thalegani et al.^35^ observed a low r_ge_ for the experimented traits, warranting that more accurate genetic information on these traits is needed to employ them for the selection of superior genotypes.

Heritability is a key factor in breeding programs, enabling plant breeders to identify plants with required heritable traits and design efficient breeding strategies to achieve expected genetic gain. Statistically, H^2^ reflects the correlation between genotypic and phenotypic expressions of a trait. It quantifies the proportion of observed variation in a trait that is attributed to genetic factors^16^. Heritability estimates for a trait range from 0 to 1. In this study, all traits exhibited low to high heritability (0.068 to 0.863) (Table 3.). The traits NC, NP, and YD displayed higher H^2^, indicating a strong relationship between phenotype and genotype for the experimented traits, further providing a fair chance of successful selection based on phenotype. High values of ‘As’ and H^2^ indicate strong genotype predictability across environments, thereby ensuring more efficient selection. When combined with the applied selection intensity (15%), these parameters provide confidence that the superior genotypes identified are not only stable but also predictable in breeding programs.

### Interpreting stability through mean vs. WAASB biplot

A WAASB biplot is divided into four quadrants where the mean performance of traits is plotted against the WAASB values. Contrary to the AMMI biplot, the WAASB biplot considers all the variations caused by GEI and helps in identifying stable genotypes^17^. The horizontal axis of the biplot represents the mean values of the traits, and the vertical axis indicates the WAASB values. The genotypes plotted on the right side of the vertical line (originating at the overall mean on the X-axis) have greater mean values than the overall mean, while those on the left side have values lower than the overall mean. The ideal/stable genotypes are those with WAASB scores near zero and mean values above the overall mean^8^.

Genotypes in Quadrant I are highly unstable and yield poorly. Quadrant II contains unstable genotypes but produces yields above the overall mean. Quadrant III features genotypes that are well adapted but yield poorly. Quadrant IV is considered important from a plant breeding perspective because it includes genotypes that are well-adapted and exhibit high performance for specific traits. The key benefit of this biplot is its usefulness in interpreting both the stability and productivity of genotypes together, which helps in identifying genotypes that can adapt well to a wider range of environments with good yielding potential.

**Fig. 2:**
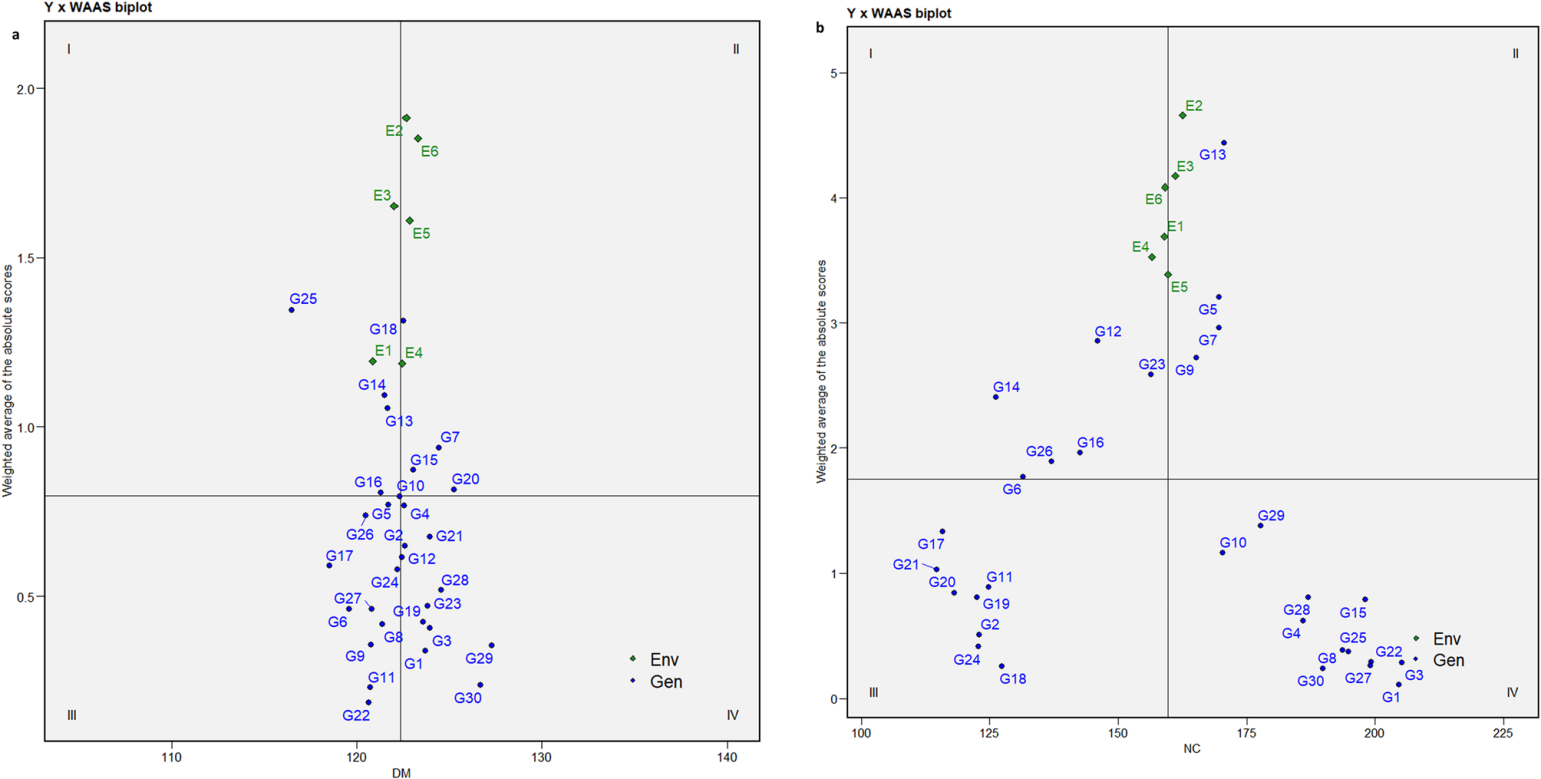

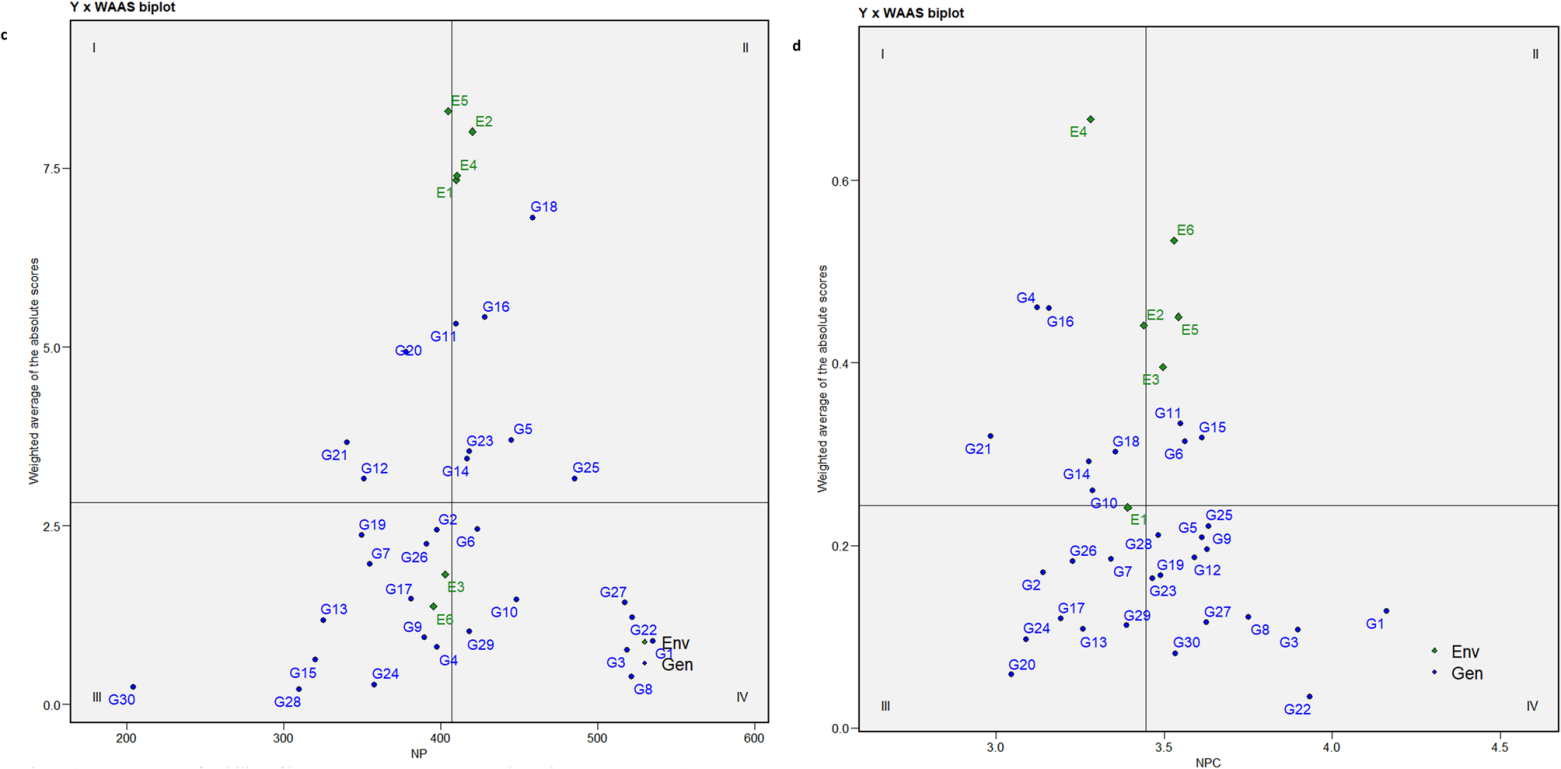

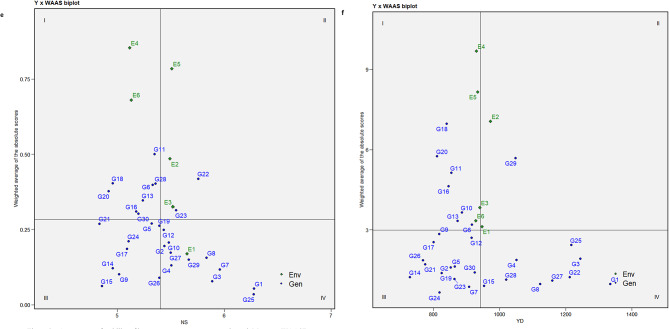
Assessment of stability of horse gram mutant genotypes through Mean vs. WAASB biplot for days to maturity -DM (**a**). Assessment of stability of horse gram mutant genotypes through Mean vs. WAASB biplot for number of cluters/plant - NC (**b**). Assessment of stability of horse gram mutant genotypes through Mean vs. WAASB biplot for number of pods/plant - NP (**c**). Assessment of stability of horse gram mutant genotypes through Mean vs. WAASB biplot for number of pods/cluster – NPC (**d**). Assessment of stability of horse gram mutant genotypes through Mean vs. WAASB biplot for number of seeds/pod - NS (**e**). Assessment of stability of horse gram mutant genotypes through Mean vs. WAASB biplot for yield/hectare - YD (**f**).

The Mean vs. WAASB biplots for all the traits are shown in Fig. 2a-f. The genotypes G25, G14, G13, and G16 for DM, G12, G23, G16, G26, and G6 for NC, G11, G20, G21, and G12 for NP, G4, G16, G21, G14, G10, and G18 for NPC, G11, G28, G6, G13, and G16 for NS, and G18, G20, and G11 for YD were observed in the first quadrant, whose WAASB values are high alas the mean values are lower the overall mean indicating their fluctuating performances for respective traits. Similarly, genotypes G18, G7, G15, and G10 for DM, G13, G5, G7, and G9 for NC, G18, G16, G23, and G25 for NP, G11, G6, and G15 for NPC, G22 and G23 for NS appeared in the second quadrant with greater trait mean than the overall mean. These genotypes perform well under favourable conditions and are suitable for cultivation in optimal growth environments. For the trait YD, G1, G8, G27, G22, G3, G25, G28 and G4 were positioned in quadrant IV, indicating their stability across environments. May not all the best-adapted genotypes be the better yielders. Among the six genotypes (Fig. 2f), G1, G3, G22, G25, G27, and G8 exhibited higher trait values than the overall mean and hence were earmarked for further utilization. Additionally, genotypes G1, G3, G27, G22, and G8 were identified as stable (quadrant IV) with exceptional performance for traits, namely NC (Fig. 2b), NP (Fig. 2c), NPC (Fig. 2d) and NS (Fig. 2e). The identification of these stable and high-performing genotypes reflects their high breeding value, as they consistently express superior performance across environments. Given the moderate to high heritability of these traits, selection based on these genotypes is expected to result in reliable and meaningful genetic gain in breeding programs. Hassani et al.^16^ in sugar beet and Gowda et al.^36^ in sweet potato utilized mean vs. WAASB biplot for selecting good yielding and stable genotypes.

### Optimizing genotype selection through MTSI

**Fig. 3:**
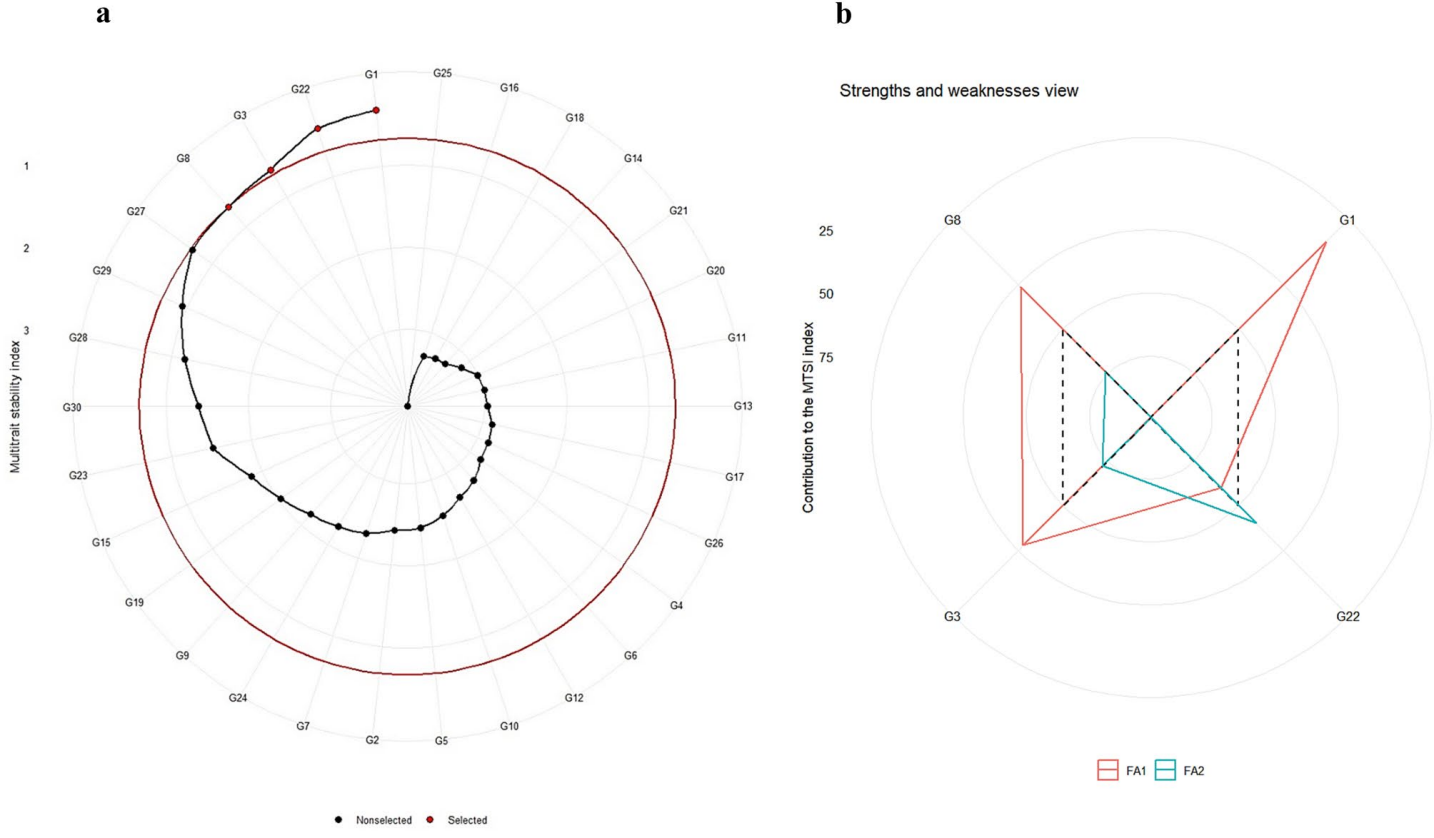
Genotype selection using multi-trait stability index plot (**a**), analysis of the strengths and weaknesses of selected genotypes, represented by the factor ratios within the multi-trait stability index (**b**).

MTSI is one of the robust techniques employed for selecting the genotypes with better performance and favourable traits. Further, it assesses the strengths and weaknesses of the selected genotypes while avoiding the problem of multicollinearity. MTSI upholds the original association structure of the dataset, while concurrently pinpointing genotypes with stability and superiority for multiple traits^20^. In MTSI estimation, initially, the principal component analysis (PCA) was performed, where the PCs with greater than 1 Eigen value were selected for factor analysis. Varimax rotation was applied to simplify and clarify the interpretation of the components. Accordingly, in the present study, two PCs were selected for further factor analysis (Table 4.). The percentage of variation explained by factor 1 (FA1) is 58.20%, and factor 2 (FA2) explained 19.60%. Similarly, Hassani et al.^16^ identified two factors in their MTSI analysis of sugar beet genotypes, but the percentages of variation explained by FA1 and FA2 were 41.76% and 36.48%, respectively. Lower MTSI values signify a closer match to the ideal genotype, while higher values indicate a greater deviation, and those genotypes are less favourable for selection. The lowest MTSI was recorded by the genotype G1 (1.192), followed by G22 (1.250), G3 (1.319), and G8 (1.841). Selection intensity is fixed based on the size and genetic variability present in the experimental population. A selection intensity of 15% was fixed to select stable genotypes by Memon et al.^37^ and Olivoto et al.^20^, which was utilized in this study to select genotypes. The genotype G1 ranked as the most stable, followed by G22, G3, and G8 for the studied traits (Fig. 3a). MTSI was employed by various researchers to distinguish superior and stable genotypes for different traits. For example, in lentils by Sellami et al.^38^ for yield, in soybeans by Zuffo et al.^39^ for drought and saline stresses, and by Hussain et al.^40^ in chickpeas for drought tolerance. Figure 3b highlights the strengths and weaknesses of the chosen genotypes, visually representing the influence of each factor on the MTSI index. The factor(s) positioned closer to the outer edge are considered the most significant contributor(s), FA1 made the highest contribution of 58.20%. FA1 comprises G1 and G3, and FA2 contributes about 19.60% and consists of G22 and G8 (Fig. 3b), indicating that these genotypes are closer to the ideal for the tested traits.

**Table 4:** Multiple trait selection index (MTSI) arrived principal components in horse gram mutant genotypes.

PC	Eigenvalue	Variance (%)	Cumulative variance (%)
PC1	3.49	58.20	58.20
PC2	1.18	19.60	77.80
PC3	0.49	8.18	86.00
PC4	0.39	6.54	92.50
PC5	0.23	3.98	96.50
PC6	0.21	3.51	100.00

*PC- Principal Components.

### Selection differential and trait improvement in selected genotypes

The selection differential (SD) is the measure of the difference between the trait mean value of the selected individuals and the population documented before selection for traits^41^. The selection gain (SG) represents the expected genetic improvement realized after selection. Reporting both parameters is important because SD indicates the extent of superiority of the chosen genotypes, while SG quantifies the actual improvement anticipated in the population following selection. Together, these measures provide a comprehensive understanding of the selection response. In that context of the examined traits, SD explains the superior performance (in percentage) of the selected genotypes compared to the overall population mean for a specific trait. SD values for NC, NPC, NP, NS, YD, and DM were 25.70, 14.20, 28.80, 9.94, 30.40 and 0.05% respectively (Table 5.), indicating that the selected genotypes outperform for YD by 30.40% which is considered significant in horse gram yield improvement programs. Further, the SD values for all the other traits are also higher than the population mean. This reflects a positive selection gain of 24.70, 10.80, 28.00, 7.81, 30.00 and 0.03% for NC, NPC, NP, NS, YD, and DM, respectively. Together with the selection differential and selection gain observed for these traits, the MTSI results indicate that selecting these genotypes will provide reliable multi-trait improvement, reflecting high breeding value and substantial expected genetic gain in the population.

**Table 5:** MTSI arrived selection differential for the biometrical traits of horse gram mutant genotypes.

Traits	FA	FA1	FA2	Xo	Xs	SD%	SG%	Sense	Goal
NC	FA1	−0.78	−0.26	160.00	201.00	25.70	24.70	Increase	100
NP	FA1	−0.89	−0.16	407.00	524.00	28.80	28.00	Increase	100
NPC	FA1	−0.59	−0.56	3.45	3.94	14.20	10.80	Increase	100
NS	FA1	−0.85	0.22	5.40	5.94	9.94	7.81	Increase	100
YD	FA1	−0.89	−0.17	942.00	1229.00	30.40	30.00	Increase	100
DM	FA2	0.00	−0.93	122.00	122.00	0.05	0.03	Increase	100
Eigenvalues		3.49	1.17	-	-	-	-	-	-
Variance (%)		58.20	19.60	-	-	-	-	-	-
Cumulative variance (%)		58.20	77.80	-	-	-	-	-	-

FA1- Factor1, FA2-Factor2, Xo - mean of genotypes, Xs - mean of selected genotypes.

SD% - selection differential in %, SG% - Selection gain in %.

DM -Days to maturity, NP -Number of pods/plant, NC -Number of clusters/plant, NPC – Number of pods/cluster, NS -Number of seeds/pod, YD -Yield/hectare.

The consolidated results arrived after analysing the METs of the 2023 and 2024 cropping seasons using YREM, BLUP, WAASB, and MTSI helped in identifying useful horse gram genotypes G1, G3, G22, G27, and G8 due to superior performance for the analysed traits.

## Conclusion

The current MET experiments (2023 and 2024 cropping seasons) utilizing various stability indices/methods (YREM, BLUP, WAASB and MTSI), was carried out to test and verify the findings of the METs conducted during the 2021 and 2022 cropping seasons. In the later METs, the stable and better-performing genotypes were identified using AMMI and GGE biplot approaches. A slight variation in the findings between METs was noticed. Based on the METs of the 2021 and 2022 seasons, the mutant genotypes G1, G27, G25, and G3 were identified as stable and best performers. However, the consolidated results arrived after employing a set of different stability approaches for the MET of the 2023 and 2024 cropping seasons; the genotypes G1, G3, G22, G27, and G8 were identified as promising. The combined results of different METs conducted during the 2021, 2022, 2023, and 2024 cropping seasons using different analytical methods (AMMI, GGE, BLUP, WAASB, MTSI), the mutant genotypes G1, G3, and G27 are earmarked as superior performers and stable genotypes, indicating their genome buffering capacity across varied environments. Both AMMI and BLUP techniques help in analysing GEI in METs, aiming to identify stable, adaptable, and productive genotypes. However, the BLUP models typically provide more reliable estimates of genotypic means, thus enhancing the prediction accuracy of genotypic performance. The variation in genotype identification G25 in the fixed effect model and G22 and G8 in the linear mixed effect model approach is attributed to (i) the sensitivity of these analytical methods to subtle GEI and (ii) year-to-year environmental weather parameter variations. This discrepancy emphasizes the need for multi-year and multi-method assessments in comprehensively evaluating genotype stability. The consistent performances across years and analytical methods confirm the stability and performance ability of G1, G3, and G27 while highlighting G25, G22, and G8 as promising candidates for further investigation.

## Materials and methods

### Genetic material and experimental design

The genetic material comprised 30 horse gram mutants (29 mutant genotypes and one parent, PAIYUR 2). The genotypic details of the experimental materials are provided in Jafarullakhan et al.^10^. The parent PAIYUR 2 was genome restructured for growth type (determinate/indeterminate), photoperiodic responsivity (photosensitive/photo-insensitive), and yield utilizing induced mutagenesis involving various combinations of gamma rays, electron beam, and EMS. The details of the combinations were described by Sudhagar et al.^42^. The genetic materials were evolved and characterized for plant breeding potentials by following prescribed plant breeding procedures from 2016 to 2019 with the funding support of the Government of India (Board of Research in Nuclear Sciences (BRNS) funded scheme. In the cropping year 2020, with the financial support of the DST (Department of Science and Technology), Government of India scheme, the variability generated through induced mutagenesis for yield-related and nutritional traits was reported by Pushpayazhini et al.^21^., from which 29 promising mutants were identified and earmarked for further evaluation. The genome plasticity of these horse gram mutants for yield potential was assessed through a multi-environment-based experiment (MEE) for two cropping years (2021 and 2022) over three environments using AMMI and GGE biplot techniques^10^. The experiment was conducted using a Randomized Block Design (RBD) with six replications at each environment, and all genotypes were included once in every replication.

Based on the stability, nutritional perspective and amino acid profiling, four mutants viz., G1, G3, G25 and G27, were earmarked as promising from the breeding perspective. To test and verify this finding, the same 30 genotypes were evaluated in other METs during the 2023 and 2024 cropping seasons, using a different set of Linear Mixed Model (LMM) methodologies suggested by Olivoto et al.^8^.

### Experiment location and data Documentation

The confirmative trial was conducted in three locations over two cropping years 2023 (E1, E2 & E3) and 2024 (E4, E5 & E6) and therefore the total study environments were considered six. The experiments were conducted during the *Rabi* seasons. The geographic distribution of the experimental locations is shown in Supplementary Fig. S4. The map was generated using QGIS software, version 3.4 (https://qgis.org)^43^. The experimental site locations and coordinates were E1&E4: the experimental farm of the Department of Pulses (11.02 °N and 76.92 °E), CPBG (Centre for Plant Breeding and Genetics), Tamil Nadu Agricultural University, Coimbatore, Tamil Nadu, E2&E5: Sugarcane Research Station, Melalathur (12.91°N and 78.87 °E), Tamil Nadu Agricultural University, Vellore, and E3&E6: a farmer’s experimental field (12.34 °N and 78.13 °E), Krishnagiri district, Tamil Nadu (which is one of the major horse gram area of Tamil Nadu). The weather parameters like temperature (maximum and minimum-°C), relative humidity (%), and rainfall (mm) prevailed for two cropping years were studied to understand the weather variability and genome plasticity of test genotypes in the study environments. The meteorological datasets are available at the official site of National Aeronautics and Space Administration (NASA POWER) (https://power.larc.nasa.gov/). Details of the soil characteristics and fertility status of experimental fields are provided in Supplementary Table 1. The test genotypes were sown in 5-meter rows, and the spacing pattern was 45 × 15 cm. All recommended cultivation practices were implemented to ensure proper crop establishment and growth. The management practice included need-based irrigation, fertilizer application (12.5 kg/ha nitrogen, 25 kg/ha phosphorus, 12.5 kg/ha potassium), and one hand weeding at 25–30 days after sowing. No plant protection measures were taken up since the crops were diseases and pests free. Ten plants per genotype were randomly selected from each replication and data were recorded on ten traits namely, day to maturity (DM), plant height (PH), days to 50% flowering (DF), number of clusters/plant (NC), number of seeds/pod (NS), number of pods/cluster (NPC), number of primary branches (NB), hundred seed weight (HSW), number of pods/plant (NP), and yield/hectare (YD) as specified by Mahajan et al.^44^. The biometrical data on DF and DM were recorded at appropriate stages, while all other data were documented after harvest and during post-harvest processing.

### Statistical analysis

#### Estimation of overall mean and multiple-trait correlation

The overall means of the studied traits across environments, along with Pearson’s correlation coefficients and correlograms indicating significance levels, were constructed for the ten traits using R Studio-METAN package, version 4.1.1 (https://cran.r-project.org/)^45^. The most interrelated traits with yield alone were further considered for analyses.

#### Yield relative to the environmental maximum (YREM) of genotypes

YREM is the index for evaluating the genotype’s yield performance relative to the best-performing genotype across environments^18^. It is calculated as $\:{Y}_{ij}\:=\:{X}_{ij}/{MAX}_{ij}$, Where Y_ij_ represents the YREM value, X_ij_ denotes the yield of a specific genotype, and MAX_ij_ refers to the maximum yield recorded by any genotype across locations. The range of YREM will be 0 to 1. The crossover GEI intensity in a MET can be ascertained using YREM. The effect of GEI crossover on yield can be calculated as 1-YREM of a genotype. YREM was calculated using Microsoft Excel 2016 (Version 2306).

#### Additive main effects and multiplicative interactions (AMMI) model

AMMI model was utilized to ascertain the extent of variability due to test genotypes (G), study environments (E), and their interaction (GEI)^46^. The additive effects of G and E were assessed using the traditional ANOVA arrived at the first stage of AMMI analysis, while the non-additive effects related to GEI interactions were analysed through Principal Component Analysis (PCA).

$$\:{Y}_{ij}=\mu\:+{g}_{i}+{e}_{j}+\sum\:_{k=1}^{n}{\lambda\:}_{k}{\alpha\:}_{ik}{\gamma\:}_{jk}+{\theta\:}_{ij}$$

Y_ij_​ represents the average performance of test genotype i (where i = 1,2,…,n) in study environment j (where j = 1,2,…,m). The general mean is denoted by µ, g_i_ ​represents the effect of test genotype i, and e_j_​ denotes the effect of study environment j. The λ_k_ ​ is the eigenvalue for the *k*th PCA axis, while α_ik_ and γ_jk_ ​ are the PCA scores for genotype i and environment j on the *k*th axis, respectively. The residual variation is denoted by θ_ij_.

#### Best linear unbiased predictor (BLUP) Estimation

BLUP is functioning based on the LMM where G and GEI were treated as random effects, and their significance was assessed using the Likelihood Ratio Test (LRT). The BLUP of the specific genotype is predicted as, $\:BLUPi=\mu\:+\widehat{{g}_{i}}\:,\:$where µ representing the grand mean and$\:\widehat{gi}$ is the genotypic effect. The genotypic effect is calculated using the formula, $\:\widehat{gi}={h}^{2}g\left(\stackrel{-}{yi}-\stackrel{-}{y}\dots\:\right)$. In this equation, h^2^g denotes the shrinkage effect for the test genotype, $\:\stackrel{-}{{y}_{i}}\:$is the mean of the *i*th genotype across all environments and, $\:\stackrel{-}{y}$ signifies the overall average across all genotypes and all environments.

#### Estimation of genetic variance components (GVC) from mixed models

The GVC estimation is essential for interpreting the BLUP results and guiding selection decisions in plant breeding. Each of these metrics contributes to the reliability, precision, and applicability of the BLUP analysis. The GVCs are coefficient of determination for GEI (R² GEI), selection accuracy (A_S_), genotype-environment correlation (r_ge_), and heritability (H^2^) were estimated following the procedures outlined by Olivoto et al.^8^. The traits with a lower R² GEI and a higher As, r_ge,_ and H^2^ are earmarked and used for genotypic selection.

#### The weighted average of absolute scores from BLUP (WAASB) biplot

The WAASBs were derived from the singular value decomposition of the BLUP matrix for exploring the GEI effects. The genotypes with lower WAASB values have greater stability^8^.

$$\:WAASBi=\frac{\sum\:_{k=1}^{p}\left|{IPCA}_{ik}\times\:{EP}_{k}\right|}{\sum\:_{k=1}^{P}{EP}_{k}}$$

Where, WAASB_i_ denotes the weighted average of absolute scores, calculated for either the *i*th environment or genotype. IPCA_ik_ is the score of *i*th environment or genotype in the *k*th IPCA. The EP_k_ is the degree of variance described by *k*th IPCA.

#### WAASBY (weighted average of absolute scores from BLUP and yield) index

The WAASBY utilizes the stability score calculated by the WAASB index, and yield performance (Y) to identify better-performing and stable genotypes. It is calculated based on the formula,

$$\:WAASBYi=\:\frac{\left(rYi\:\times\:\:\theta\:y\right)+(rWi\:\times\:\:\theta\:s)}{\theta\:y+\:\theta\:s}$$

Where, rYi and rWi denote the rescaled values (0–100) for the response variable and WAASB, respectively. θy and θs represent the weights assigned to performance and stability. In this case, θy = 65% and θs = 35%, prioritizing mean performance over stability^8^.

#### Multi trait stability index (MTSI)

The MTSI is an important tool in the LMM framework which helps in selecting superior genotypes by considering multiple traits together at analysis. It considers the interrelationship between multiple traits. This makes the selection process more comprehensive and potentially more accurate. It is based on the factor analysis comprising three steps viz., (1) Multi-trait selection using genotype-ideotype distance, (2) Ideotype planning, and (3) MTSI calculation. An ideotype represents the optimal plant type, characterized by the desirable traits for thriving in a given environment. The genotype scores were calculated using WAASBY means. The ideotype scores were arrived at based on the assumption that an ideotype achieves the maximum WAASBY value of 100 for each of the experimented traits. Consequently, the MTSI was computed according to the formula,

$$\:{MTSI}_{i}={\left[\sum\:_{f=1}^{f}({F}_{ij}-{F}_{j}{)}^{2}\right]}^{0.5}$$

MTSI represents the multi-trait stability index for the *i*th genotype. The F_ij_ denotes the *j*th trait score of the *i*th genotype. The Fj corresponds to the *j*th trait score of the ideal genotype^20^. A lower MTSI score denotes a higher level of stability for a genotype.

#### Selection differential (SD%)

SD is used to quantify the effectiveness of selection by measuring the difference in the mean values of the selected individuals and the original population. It is computed using the formula,

$$\:SD{\%}=\left(\frac{{X}_{s}-{X}_{0}}{{X}_{0}}\right)\times\:100$$

Where, Xs and X_0_ are the mean performance value of the selected individuals and initial mean value before selection, respectively.

#### Statistical software

All the statistical analyses pertaining to LMM were performed using R Studio with the METAN package, version 4.1.1 (https://cran.r-project.org/)^45^.

## Supplementary Information

Below is the link to the electronic supplementary material.

Supplementary Material 1

## Data Availability

All data documented in these experiments are published in this article.

## Declarations

### Plant experiments

All the experiments in this manuscript were conducted on an Indigenous and cultivated horse gram species (*Macrotyloma uniflorum* Lam. Verdc.) by following relevant institutional and national guidelines and legislation.
