# Supplementary material for: Multi-model-based validation of multi-environment trial results in horse gram (Macrotyloma uniflorum Lam. Verdc.)
Source: Sci Rep. 2025 Nov 21;15:41266. doi: 10.1038/s41598-025-25093-2 (PMC12638885; doi:10.1038/s41598-025-25093-2)
Supplement: Supplementary file 1 — Supplementary Material 1 [file 41598_2025_25093_MOESM1_ESM.doc]

**Multi-model-based validation of multi-environment trial results in horse gram (*Macrotyloma uniflorum* Lam. Verdc.)**

Sudhagar Rajaprakasam^1*^, Sumaiya Sulthana Jafarullakhan^1^, Vaishnavi Vijayakumar^1^, Naaganoor Ananthan Saravanan^2^, Sivakumar Rathinavelu^3^, Balaji Kannan^4^, Vanniarajan Chockalingam ^5^, Raveendran Muthurajan^6^, & Selvaraju Kanagarajan^7^

**Supplementary figures**


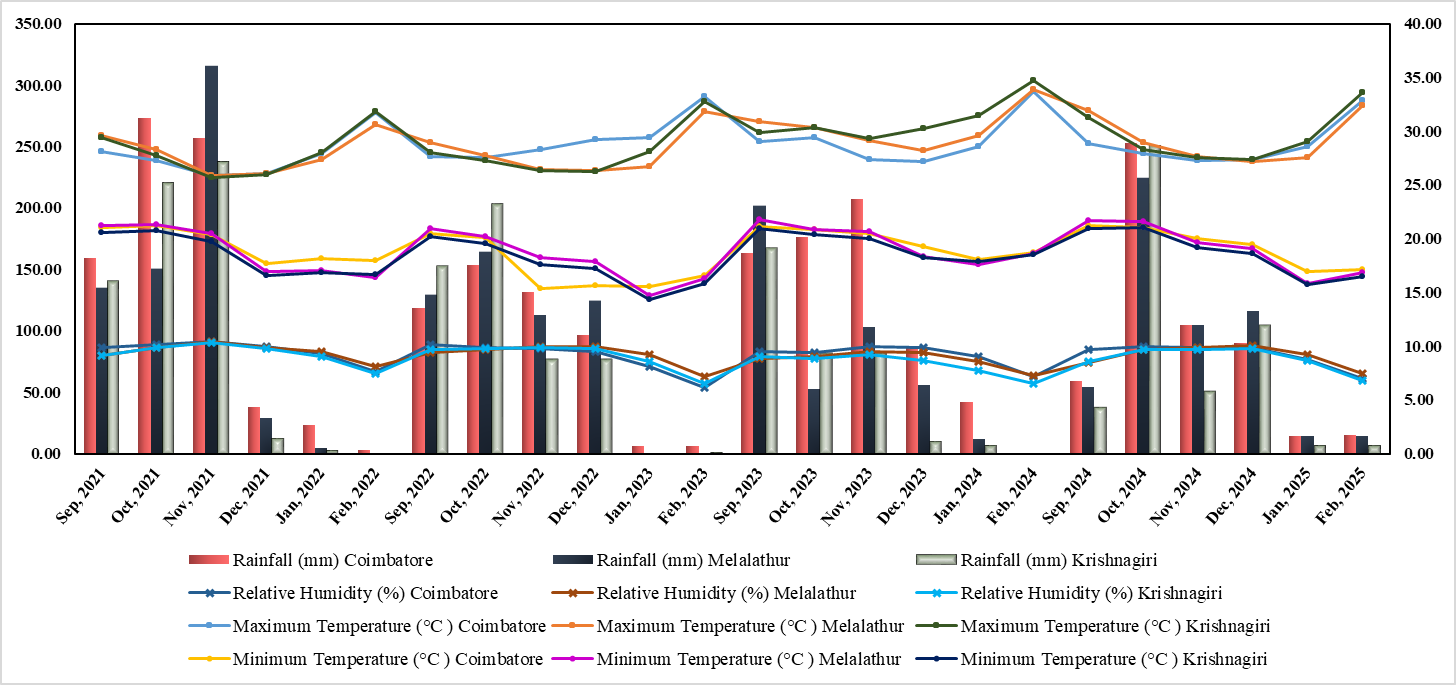


Supplementary Figure S1. Monthly weather variations across study environments highlighting Rainfall (RF), Temperature maximum (T.max) and minimum (T.min), and Relative humidity (RH) for the cropping years 2021, 2022, 2023, and 2024 (*Rabi* season: September to February). Source: <https://power.larc.nasa.gov/>


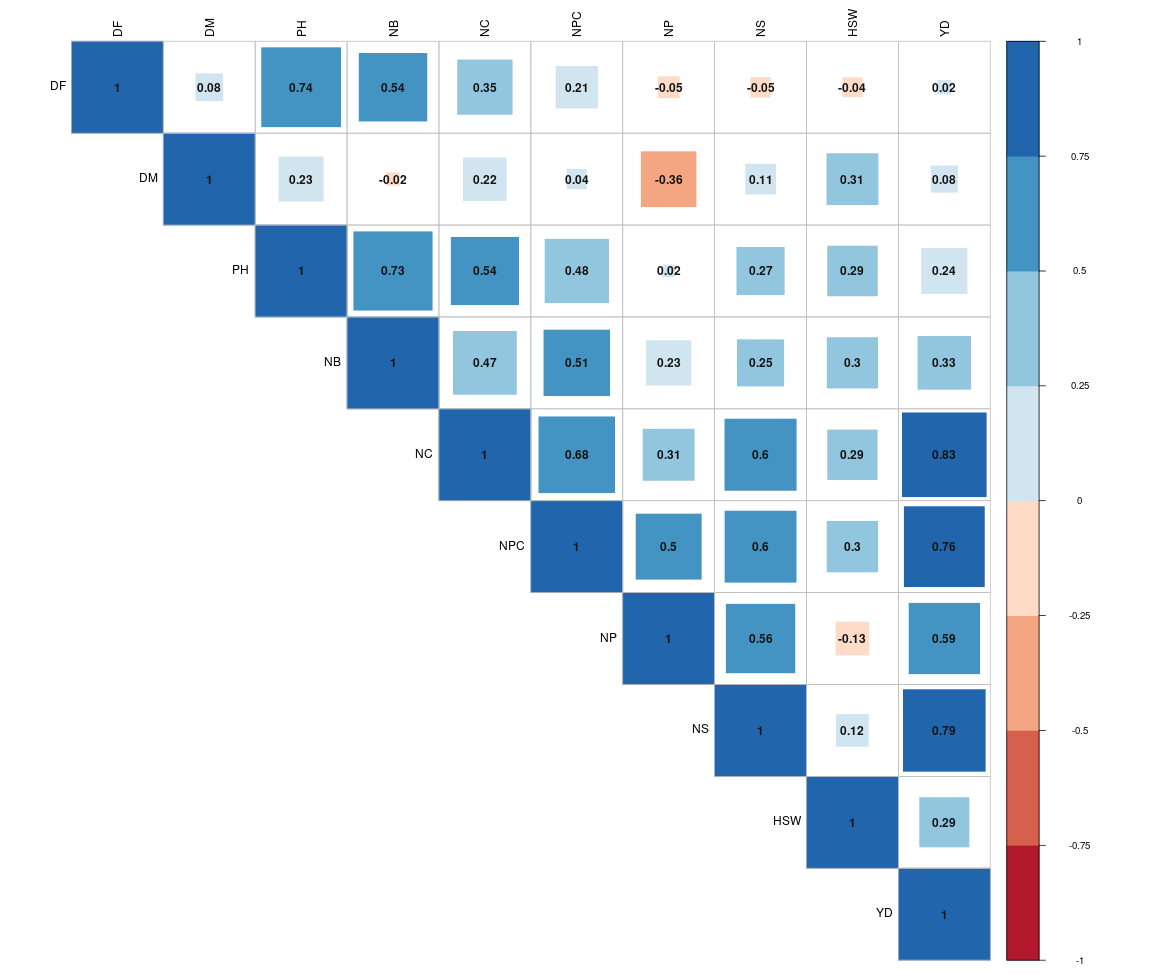


Supplementary Figure S2. Correlogram for different biometrical traits of horse gram genotypes [day to maturity (DM), plant height (PH), days to 50% flowering (DF), number of clusters/plant (NC), number of seeds/pod (NS), number of pods/cluster (NPC), number of primary branches (NB), hundred seed weight (HSW), number of pods/plant (NP), and yield/hectare (YD)]


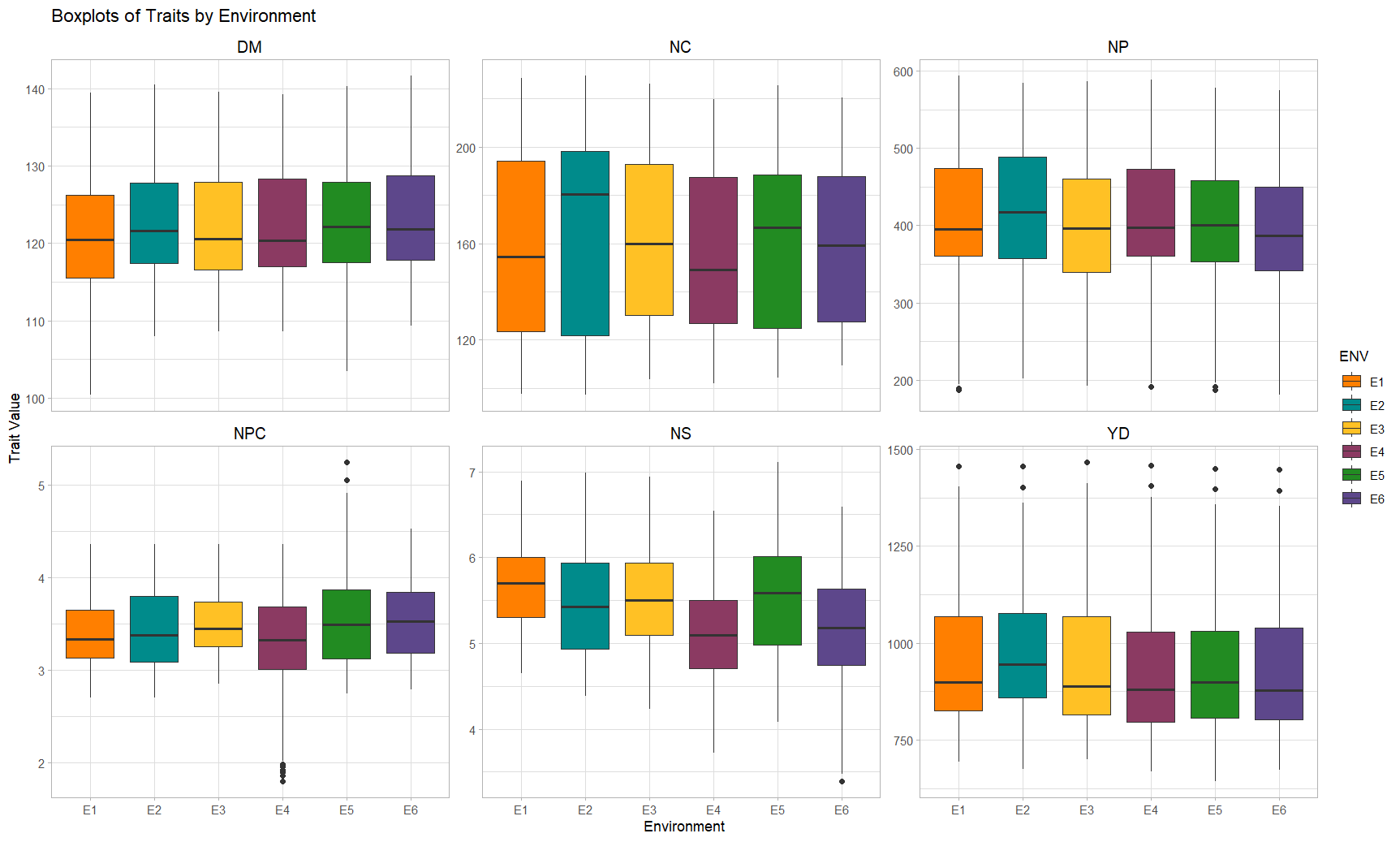


Supplementary Figure S3. Box plot showing the trait variation in horse gram genotypes [days to maturity -DM, number of clusters/plant -NC, number of pods/plant -NP, number of pods/cluster –NPC, number of seeds/pod -NS, and yield/hectare-YD, ENV-Environment (E1, E2, E3, E4, E5, & E6)]


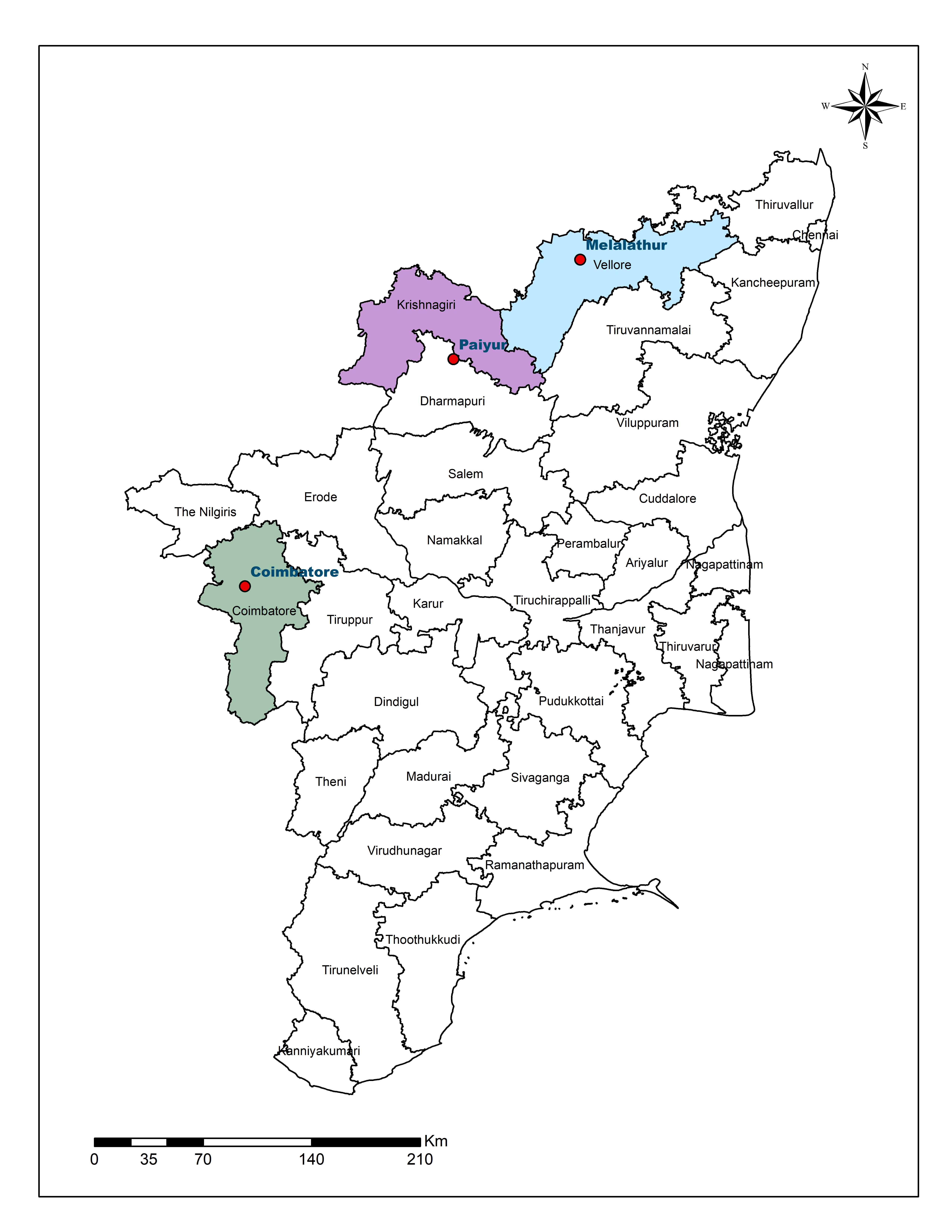


Supplementary Figure S4. Geographical map of study environments [E1&E4: the experimental farm of the Department of Pulses (11.02 °N and 76.92 °E), CPBG (Centre for Plant Breeding and Genetics), Tamil Nadu Agricultural University, Coimbatore, Tamil Nadu, E2&E5: Sugarcane Research Station, Melalathur (12.91°N and 78.87 °E), Tamil Nadu Agricultural University, Vellore, and E3&E6: a farmer’s experimental field (12.34 °N and 78.13 °E), Krishnagiri district, Tamil Nadu]. The map was generated using QGIS software version 3.4 ([https://qgis.org](https://qgis.org/).)

**Supplementary Table**

Supplementary Table 1. Soil characteristics and fertility status of experimental fields

| **Environments** | **pH** | **Electrical Conductivity (EC) dS/m** | **Organic Carbon (OC) g/kg** | **Available Nitrogen**  **(kg/ha)** | **Available Phosphorus**  **(kg/ha)** | **Available Potassium**  **(kg/ha)** |
| --- | --- | --- | --- | --- | --- | --- |
| Experimental farm of the Department of Pulses, Coimbatore, Tamil Nadu  E1&E4 | 8.75 | 0.53 | 3.3 | 224 | 17 | 610 |
| Sugarcane Research Station, Melalathur, Tamil Nadu  E2&E5 | 7.77 | 0.27 | 3.7 | 112 | 9 | 156 |
| A farmer’s experimental field, Krishnagiri,  Tamil Nadu  E3&E6 | 7.69 | 0.06 | 4.1 | 238 | 24.8 | 177 |
